# Pericardial Corns

**Published:** 1900-02-03

**Authors:** 


### PERICARDIAL CORNS.

In a recent clinical lecture at the Polyclinic Sir William Broadbent[1] dealt with a case in which there was an extracardial murmur, due apparently to a condition which he described as "corns" of the pericardium. The patient was a telegraphist, aged 43, who had always enjoyed good health, and had never had rheumatic fever, or, indeed, any serious illness. His illness had commenced a couple of months before with an attack of vertigo with pains in the limbs. For a fortnight he had repeated attacks, usually occurring at night, and accompanied with pain in the region of the heart and with difficulty of breathing, which had obliged him to sit up in bed. Returning to work brought on another attack, so that altogether he had had to rest for a month, after which time he had been able to resume his occupation. On examination a faint cardiac murmur was found, presystolic in time, and running over into the systole, but this was not constant. The question arose whether this bruit had anything to do with the attacks of vertigo. Examination showed a pulse of high tension, but no enlargement of the heart, a short first sound at the apex, and a murmur more or less distinct over the right ventricle. This murmur gave the impression of being extra-cardial, and resembled the "scratch" produced by patches upon the pericardium. It disappeared on firm pressure. The vertigo was probably of gastric origin, and had no connection with the heart. "The principal interest of the case," said Sir William Broadbent, "lies in the scratch murmur best heard close to the ensiform cartilage. This form of murmur is much commoner than is usually believed, and leads to many errors of diagnosis. Many a candidate for the army medical examinations is rejected quite unnecessarily on account of just such a murmur. The patches are not the result of any form of pericarditis, show no tendency either to spread or to disappear, and may last a whole lifetime without giving rise to any mischief whatever. In fact, they may be best characterised as 'corns' of the pericardium."

[1] The Polyclinic, January, 1900.

